# Oral administration of *Lactobacillus casei* DG® after ileostomy closure in restorative proctocolectomy: a randomized placebo-controlled trial (microbiota and immune microenvironment in pouchitis -MEP1)

**DOI:** 10.1080/19490976.2024.2423037

**Published:** 2024-11-01

**Authors:** Imerio Angriman, Melania Scarpa, Edoardo Savarino, Ilaria Patuzzi, Alessandra Rigo, Andromachi Kotsafti, Astghik Stepanyan, Elisa Sciuto, Francesco Celotto, Silvia Negro, Antonino Caruso, Cesare Ruffolo, Romeo Bardini, Salvatore Pucciarelli, Brigida Barberio, Gaya Spolverato, Fabiana Zingone, Renata D’Incà, Ignazio Castagliuolo, Marco Scarpa

**Affiliations:** a General Surgery 3 Unit, Department of Surgical, Oncological and Gastroenterological Sciences, DiSCOG, University of Padova, Padova, Italy; b Immunology and Molecular Oncology Diagnostics, Veneto Institute of Oncology IOV-IRCCS, Padova, Italy; c Gastroenterology Unit, Department of Surgical Oncological and Gastroenterological Sciences DiSCOG, University of Padova, Padova, Italy; d Eubiome srl, Padova, Italy; e Department of Molecular Medicine DMM, University of Padova, Padova, Italy

**Keywords:** Probiotics, microbiota, pouchitis, ileal pouch-anal anastomosis, inflammatory bowel disease

## Abstract

Pouchitis is an idiopathic inflammatory disease that may occur in ileal pouches, and it can lead to ileal pouch failure. This was a single-center, randomized, double-blinded, placebo-controlled trial that assessed the effect of *Lactobacillus casei* (*L. casei*) DG®, a probiotic strain, on the ileal pouch mucosa to determine the crosstalk between microbiota and mucosal immune system. Fifty-two patients undergoing restorative proctocolectomy were recruited and randomly assigned to receive a daily oral supplementation of *L. casei* DG® (*n* = 26) or placebo (*n* = 26) for 8 weeks from the ileostomy closure (T0) to a pouch endoscopy after 8 weeks (T1) and 1 year (T2). Ileal pouch mucosa samples were collected at T0, T1, and T2. At T1, the *L. casei DG*®-supplemented group showed a significant reduction of inflammatory cytokines levels compared to T0 baseline levels in the pouch mucosa, whereas in the placebo group cytokines levels resulted stable. In conclusion, probiotic manipulation of mucosal microbiota by *L. casei DG*®-supplementation after stoma closure in patients who underwent restorative proctocolectomy has a beneficial impact on the ileal pouch microenvironment. Registration number: NCT03136419 (http://www.clinicaltrials.gov).

## Introduction

Approximately, 20–25% of ulcerative colitis (UC) patients undergo restorative proctocolectomy with ileal pouch-anal anastomosis.^1^ This procedure can lead to good functional results since most patients are fully continent, have six bowel movements per day on average, and can defer a bowel movement until convenient.^2^ Moreover, the long-term quality of life after ileal pouch surgery is excellent, increasing after the first two years after surgery, without any deterioration thereafter.^3^ Nevertheless, the surgery is, directly or indirectly, associated with various structural, inflammatory, and functional adverse sequelae.^4^ Pelvic sepsis and poor function are the main reasons for the later failure of ileal pouch-anal anastomosis^5^ but there are also less dramatic and more frequent complications.

Indeed, approximately half of the patients with UC who undergo restorative proctocolectomy with ileal pouch-anal anastomosis will subsequently develop pouchitis and, among those patients, one-fifth will have chronic pouchitis.^6^ Pouchitis is an idiopathic inflammatory disease that may occur in ileal pouches and it is the most frequent complication after restorative proctocolectomy.^7^ Several studies showed altered microbiota and innate immunity relationships in pouchitis.^8–10^ In patients who experience recurrent episodes of pouchitis that respond to antibiotics, the AGA suggests using probiotics to prevent recurrent pouchitis.^7^ Moreover, a recent meta-analysis of trials on probiotics revealed significantly lower odds of pouchitis with the use of probiotics concluding that probiotics are effective in preventing pouchitis after restorative proctocolectomy while antibiotics are likely effective in treating active pouchitis.^11^

Recent studies suggest that *Lactobacillus casei (L. casei)* can effectively relieve dextran sodium sulfate (DSS)-induced ulcerative colitis mouse model.^12^ Moreover, *L. casei Zhang* supplementation inhibited the expression of gamma-aminobutyric acid type A receptor in mice with colitis, promoted the proliferation and renewal of colon epithelial cells, and alleviated intestinal microflora disorder in mice.^13^ A combination of *L. casei Zhang*, *Lactobacillus plantarum*, and *Bifidobacterium animalis* subsp. *lactis* obtained a significantly greater magnitude of reduction observed in the UC disease activity index compared with the placebo group, protecting from diminishing the microbiota diversity and richness.^14^ At a mucosal level, conditioning intestinal dendritic cells with probiotic strain *L. casei Shirota* in UC partially restored their normal function indicated by reduced Toll-like receptor 2/4 expression and restoration of their ability to imprint homing molecules on T cells and to generate interleukin-22 production by stimulated T cells.^15^ Moreover, we observed that rectal administration of *L. casei DG®* modifies flora composition and Toll-like receptor expression in the colonic mucosa of patients with UC.^16^ However, no study has ever investigated the *L. casei DG®* effect on pouchitis.

Therefore, we aimed to perform a double-blind, placebo-controlled trial of *L. casei DG®* therapy vs. placebo starting at the time of ileostomy closure to determine how an early microbiota manipulation in the pouch may affect inflammatory cytokines production in the pouch mucosa.

## Materials and methods

### Study design and participants

**Figure 1.**
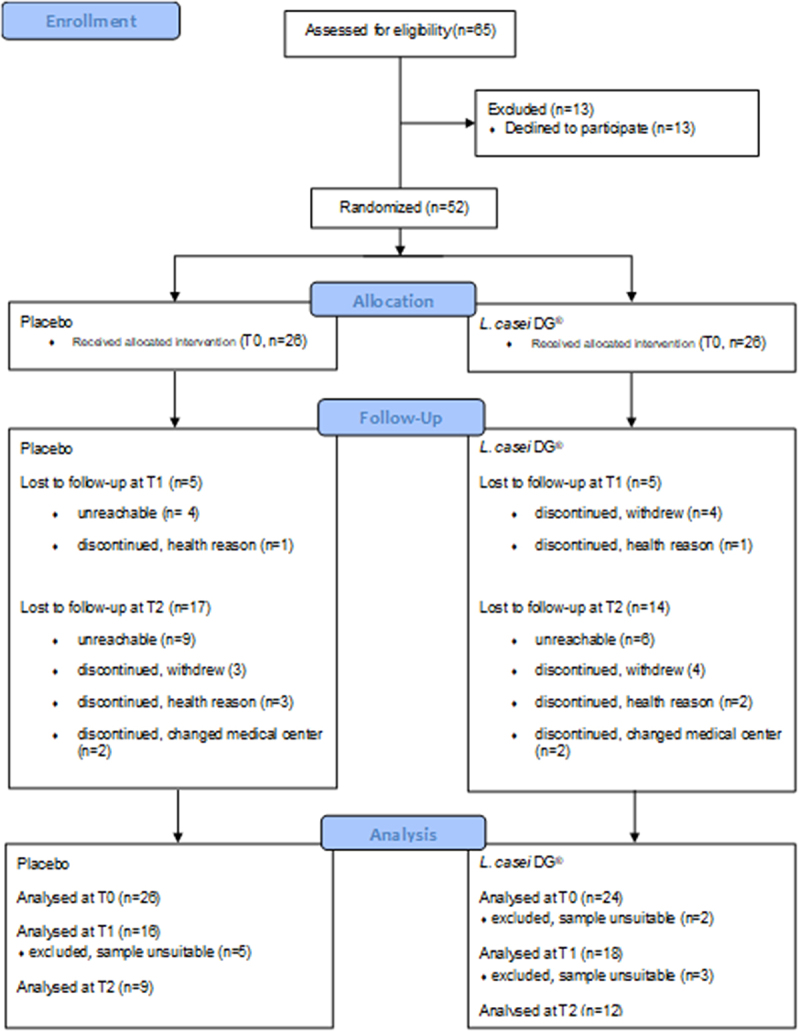
CONSORT flow diagram for enrollment, allocation, follow-up, and analysis.

This study was a single-center, randomized, double-blinded, placebo-controlled clinical trial among consecutive patients who underwent restorative proctocolectomy in the General Surgery Unit at the University of Padova from October 2016 to September 2022, following the standards of reporting trials (CONSORT) guidelines. The eligible patients were randomly assigned to probiotic or placebo groups at ileostomy closure in a 1:1 ratio in a blinded fashion with a randomization table by an external researcher. Staff and investigators did not have access to the allocation list and remained blinded until analysis of the primary outcome were completed. Patients were asked to undergo pouch endoscopy with mucosal biopsies at the following times: a) at ileostomy closure (T0), b) 2 months after ileostomy closure (T1), c) 12 months after ileostomy closure (T2) (or before, in case of overt pouchitis) (Figure 1).

### Ethical approval

The trial was approved by the ethics committee of the Azienda Ospedaliera di Padova, Italy (Project ID MEP1), registered with ClinicalTrials.gov (NCT03136419), and performed according to the principles of the Declaration of Helsinki. Each patient was provided with detailed information about the study aims and methodology and was asked to give written, informed consent before enrollment.

### Eligibility criteria

*Inclusion Criteria*: consecutive patients with UC who underwent restorative proctocolectomy with ileal pouch-anal anastomosis and who attended our outpatient clinic for routine endoscopic and clinical follow-up.

*Exclusion Criteria*: Patients with cuffitis (inflammation of the rectal mucosa remnant) or Crohn’s disease of the pouch (with perianal fistulae or with inflammation of the afferent ileal limb), as well as patients who received antibiotic or probiotic therapy during the previous 30 days were excluded from the study.

### Interventions

The probiotic supplement, as well as the placebo, was provided by SOFAR S.p.A/Alfasigma S.p.A. *Enterolactis®* Plus is a commercial dietary supplement containing 24 billion *Lactobacillus casei* DG® (*Lacticaseibacillus paracasei*DG I1572, DSM 34,154) per capsule. The excipient was microcrystalline cellulose. The control group received placebo capsules identical to *Enterolactis®* Plus in shape, size, taste, smell but lacked any microorganism content and included additional microcrystalline cellulose to replace the probiotic. Patients were instructed to take a capsule daily for 8 weeks after ileostomy closure. All patients continued their routine treatments during the study. *L. casei* DG® supplementation is just a diet integrator and not a proper drug so it was not necessary a formal safety assessment.

### Outcomes

*Primary Outcome*: the inflammatory cytokines (IL-1ß, IL-6, TNF-α) levels in the ileal mucosa at T1 (8 weeks after ileostomy closure).

*Secondary outcomes*: Relative abundance of bacterial taxa at T1; systemic and local inflammatory status at T2; histological inflammatory severity at T1; activation status of macrophages, dendritic cells and infiltrating lymphocytes at T1; the number and the severity of the pouchitis episodes defined as Pouchitis Disease Activity Index (PDAI)> 7 at T2 (in the 12 months after ileostomy closure). We further extended the number and severity of pouchitis episodes at the entire follow up.

### Clinical measures

Disease severity was assessed based on clinical, endoscopic, and histological criteria and PDAI score.^5^ Based on histological and endoscopic acute inflammation and clinical symptoms, patients with a total PDAI > 7 were classified as having acute pouchitis, if symptoms lasted less than 4 weeks and they responded to antibiotics, or chronic pouchitis, if symptoms lasted more or they did not respond to antibiotics anymore. Systemic and local inflammatory states were assessed at each experimental timeline by erythrocyte sedimentation rate (ESR), white blood cell count (WBC), platelets blood count (PLT), CRP, and fecal lactoferrin, respectively. ESR was measured by the Westergren method. CRP was detected by immuno-nephelometry (normal: <6 mg/l; pathological >6 mg/l). Total protein and albumin were assessed with the biuret method. WBC, platelet counts, and hemoglobinemia were obtained with standard full blood cell count. Fecal calprotectin was dosed by an ELISA test that uses rabbit polyclonal antibodies specific for human calprotectin on frozen stool samples.

### Endoscopy

Patients underwent endoscopy that included a careful examination of the afferent loop and the pouch as well as the collection of eight random biopsies from the pouch mucosa: two samples for microbiota characterization, two for conventional histology, two for molecular biology and two for flow cytometry. Samples for microbiota characterization and molecular biology were snap frozen and stored at −80°C and those for flow cytometry were collected in phosphate-buffered saline (PBS) and immediately processed for further analysis.

### Patients’ follow-up

After ileostomy closure patients were followed in the outpatients’ clinics every three months for physical examination and full blood count, renal function, calprotectin and serum ions assessment for the first year and after once a year or more often in case of problem such as pouchitis. During the visit general health status, bowel function (number of stools, consistency, presence of blood, urgency, soiling) and quality of life were recorded. Moreover, patients enrolled in the MEP1 trial underwent a pouch endoscopy at 2 months (T1) and at 12 months (T2) after the ileostomy closure.

### Histological evaluation

For routine histological examination, two biopsies were fixed in 4% PFA for 24 hours, then dehydrated and embedded in paraffin, and sections (5 μm thick) were cut and subjected to standard hematoxylin/eosin (H&E) stain. To quantify inflammatory severity, we used the Floren et al. score.^17^

### Multiplex immunoassay

Biopsies for cytokine immunoassay were homogenized mechanically in 100-µL PBS (pH 7.4). Cellular debris was removed subsequently by centrifugation (13000 g for 10 min at 4°C) and the clear supernatant was collected for analysis. Pierce BCA Protein Assay Kit (Thermo Scientific) was used for determining the protein concentration of lysates. Mucosal levels of IL-1β, IL-6, IL-10 and TNF-α were determined with the multiplexed immunometric assay in Luminex® technology (High-Plex Luminex assays).

### Flow cytometry

Ileal pouch mucosa samples were washed in Hanks’ Balanced Salt Solution containing 10 mm DTT and were digested to obtain single-cell suspensions. Freshly isolated cells (10^5^) were stained in PBS/2% FBS with appropriate combinations of FITC- and PE-conjugated antibodies. Single-cell suspensions were subjected to flow cytometry to determine the proportion of activated (CD40 and CD80 expression) dendritic cells (CD1a+), macrophage (CD163+), and T cells (CD69 expression on CD4+ and CD8+ positive lymphocytes). The specific antibodies used are summarized in Supplementary Table S1.

### DNA extraction and 16S rRNA gene amplicon sequencing

Pouch biopsies were subjected to 30 min of lysis at 37°C using lysis buffer containing lysozyme (20 mg/ml, Sigma-Aldrich), followed by DNA extraction by using the Maxwell® RSC Fecal Microbiome DNA Kit following the manufacturer’s instructions (Promega, Madison, Wisconsin) on a Maxwell® RSC 48 Instrument (Promega). The sequencing protocol was performed at BMR Genomics Srl. Briefly: V3–V4 regions of 16S rRNA gene were amplified using the primers Pro341F: 5′-CCTACGGGNBGCASCAG-3′ and Pro805R: Rev 5′-GACTACNVGGGTATCTAATCC-3′.^18^ Primers were modified with forward overhang: 5′-TCGTCGGCAGCGTCAGATGTGTATAAGAGACAG [locus-specific sequence]-3′ and with reverse overhang: 5′-GTCTCGTGGGCTCGGAGATGTGTA TAAGAGACAG [locus-specific sequence]-3′ necessary for dual-index library preparation, following Illumina protocol. Samples were normalized, pooled, and run on Illumina MiSeq with a 2 × 300 bp approach. The data that support the findings of this study have been deposited in the NCBI Sequence Read Archive (SRA) database under accession number PRJNA1130449.

### Bioinformatic analysis

Forward and reverse reads were preprocessed and analyzed using the Quantitative Insights into Microbial Ecology pipeline (QIIME2, version 2022.8).^19^ After preprocessing, the amplicon sequence variant (ASV) table was constructed using a de novo approach using the DADA2 bioinformatic plugin.^20^ The taxonomic assignment of each ASV was performed using the Greengenes database (version 13_8)^21^ and a Naive Bayes classifier trained on the target region selected for the present study (V3-V4) to achieve superior accuracy in taxonomic classification. Alpha (Richness, Pielou, and Shannon indices) and beta (Bray-Curtis, and Jaccard, Weighted and Unweighted Unifrac measures [data not shown]) diversity were calculated for microbial community diversity analysis applying a rarefaction level equal to 15,000. This cutoff was chosen after verification (using a rarefaction plot) that it was placed after each rarefaction curve had reached its plateau. Additionally, beta diversity measures were used for ordination analysis with the PCoA technique. Alpha diversity analysis was performed via QIIME2 dedicated plugins and graphically rendered in R (version 4.1.0), while beta diversity calculation and ordination plot production were performed in R using phyloseq (version 1.36.0) and vegan (version 2.5–7) packages. For the latter task, data were previously normalized using the GMPR tool (version 0.1.3)^22^ to allow for robust comparison between samples. Differential abundance analysis at the species levels was performed using ANCOMBC package (v 1.2.2).^23^

### Sample size calculation

The primary outcome of this study was to evaluate eight weeks after ileostomy closure how the inflammatory cytokines levels in the ileal pouch mucosa of patients were impacted by taking probiotic supplements when compared to placebo. Set a level of statistical significance at 0.05, a power at 0.80, and standardized effect size at 1 (as suggested by our previous studies ,^8–10^ the consequent sample size required for comparison was calculated as 16 patients for each group. In conclusion, at least 32 patients had to be enrolled in this study.

### Statistical analysis

Outcomes were analyzed according to the intention-to-treat principle, in which all patients randomly assigned to one of the treatments are analyzed together, regardless of whether they completed or received that treatment, to preserve randomization. Statistical analysis was performed using Windows Microsoft Excel and Statistica 7.1 (StatSoft, Inc.) software. Continuous data were expressed as median (range); dichotomic data were expressed as frequency and proportion. Non-parametric tests were used: Mann–Whitney U test for continuous variables and Fisher’s exact test for dichotomous ones. Statistical significance was set at *p* < 0.05.

## Results

### Patients’ enrollment and randomization

The study enrollment started on 2016–10–31 and lasted until 2022–9–30. In this period, 65 consecutive patients underwent restorative proctocolectomy in our department and were asked to participate in the study. Fifty-two patients accepted, and were randomized to receive a daily oral supplementation of *L. casei* DG® or placebo for 8 weeks from the ileostomy closure (T0: 26 patients in L. casei DG® group vs. 26 patients in the placebo group) to a pouch endoscopy after 8 weeks (T1, 21 patients in L. casei DG® group and 21 in the placebo group) and to a pouch endoscopy after 12 months after ileostomy closure (T2, 12 patients in L. casei DG® group and 9 in the placebo group). No patients, among those who completed at least T1 step, missed any treatment. The patients flow and the number and reason of the drop out at each stage of the study are shown in Figure 1 and patients’ characteristics are shown in Table 1.

**Table 1.** Patients’ characteristics at baseline.

	Placebo	*L. casei* DG®	*p value*
Gender (female/male)	F = 11 M = 14	F = 5 M = 21	0.13
Age at study enrollment (years)	54,5 (42.5–65.25)	47.5 (41,75–60.25)	0.129
Age at diagnosis (months)	456 (408–516)	408 (348–576)	0.318
Disease duration (months)	96 (36–180)	90 (48–168)	0.212
Indication to restorative proctocolectomy	Unresponsive to therapy = 18	Unresponsive to therapy = 19	0.999
	Dysplasia or cancer = 4	Dysplasia or cancer = 4	
	Severe colitis = 4	Severe colitis = 3	
Harvey–Bradshaw Activity Index	10 (IQR: 5–15)	10 (IQR: 8.25–12.5)	0.9761
Stool frequency	5 (IQR: 4–11)	7 (IQR: 5.5–11)	0.2891
Antibiotic use	none	none	N.S.
Probiotic use	none	none	N.S.
Anti-TNFalpha use	11	16	0.2668
Vedolizumab use	8	9	0.9999

### Primary outcome

**Figure 2.**
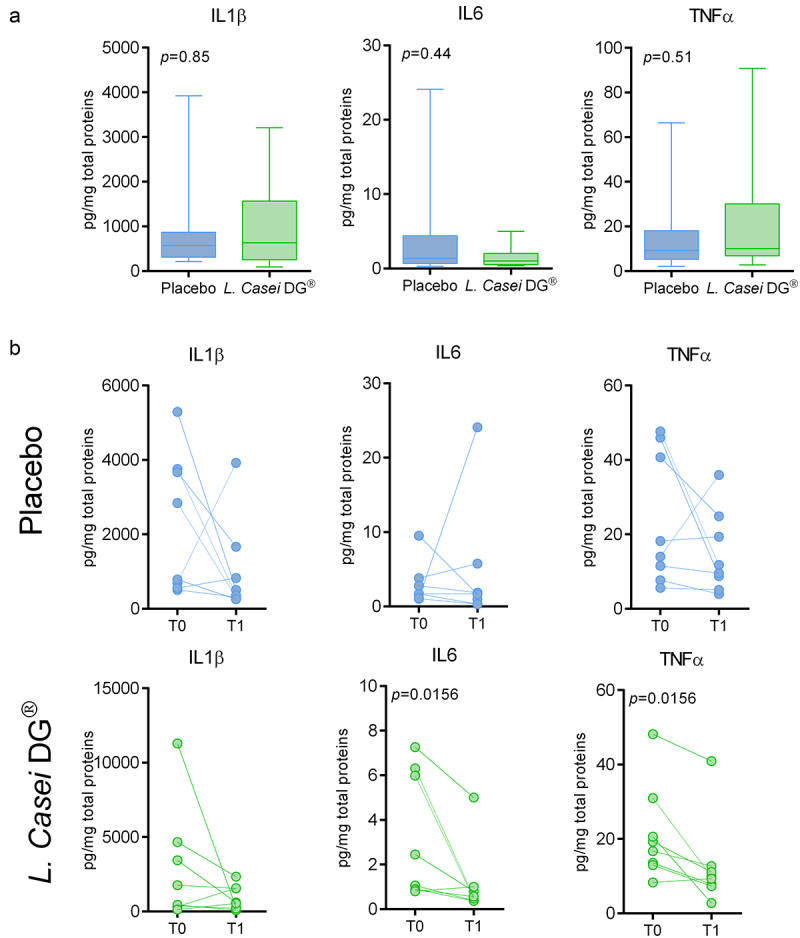
Inflammatory cytokines network in the pouch. (a) IL1β, IL6, and TNFα levels within pouch mucosa at T1 in the two arms of the study. (b) IL1β, IL6, and TNFα levels within pouch mucosa at T0 and T1 in the two arms of the study.

There was no statistically significant difference in the mucosal levels of IL1β, TNFα, IL6, and IL10 as detected by multiple immunoassays at T1 in the two treatment groups (Figure 2a and Supplementary Figure S1). However, analyzing the variation from baseline levels for each group, in the *L. casei DG*® group we observed a significant reduction at T1 in TNFα and IL6 mucosal levels (*p* = 0.0166 and *p* = 0.0156, respectively) compared to T0 baseline, while in the placebo group the inflammatory cytokines levels did not significantly change (Figure 2b). IL10 mucosal levels and variation in the different steps of the study are shown in Supplementary Figure S1.

### Secondary outcomes

#### *Effect of* L. casei *DG® supplementation on the mucosal immune microenvironment*

**Figure 3.**
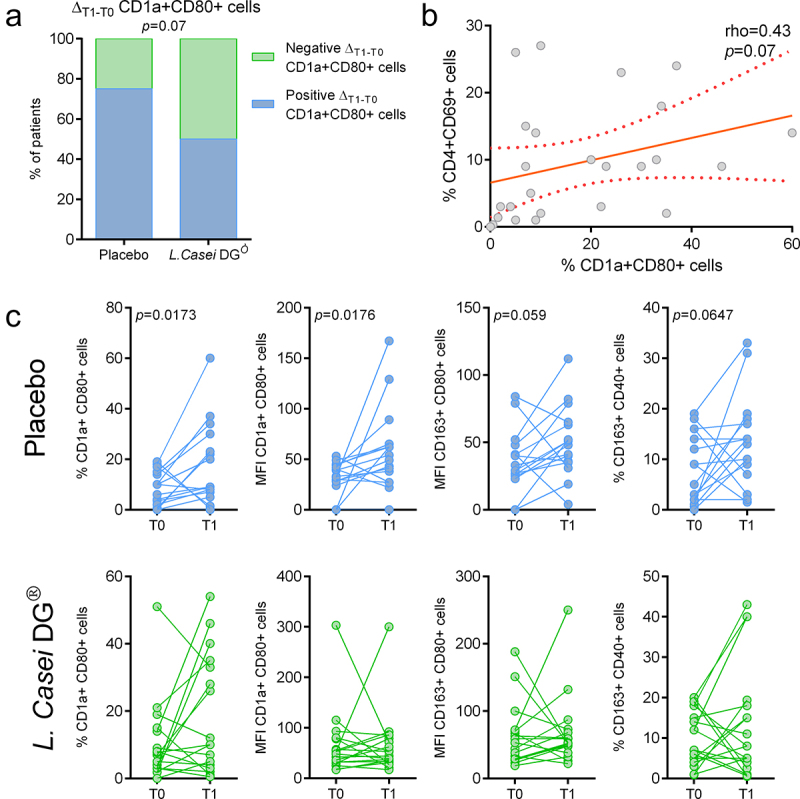
Cells subpopulations within pouch mucosa. (a) Frequency of patients with positive and negative delta T1-T0 of activated dendritic cells in the two arms of the study. (b) Correlation between activated dendritic cell rate and activated T helper lymphocytes rate. (c) CD1a+CD80+ cells rate and MFI, CD163+CD80+ cells MFI, and CD163+CD40+ cells rate in pouch mucosa at T0 and T1 in the two arms of the study.

The *L. casei DG®* supplemented group tended to have a higher frequency of patients with a decrease of CD1a+CD80+ activated dendritic cells rate in the ileal pouch mucosa in the T0-T1 time frame compared to the placebo group (*p* = 0.07) (Figure 3a). Moreover, at T1, CD1a+CD80+ dendritic cells rate tended to correlate with CD4+CD69+ activated T helper lymphocytes (rho = 0.43, *p* = 0.07) (Figure 3b). Besides, analyzing the variation from baseline levels for each group (Figure 3c), in the *L. casei DG*® group we observed stable levels of activated dendritic cells and macrophage infiltration in the pouch mucosa. On the other hand, in the placebo group, we observed a significant increase at T1 in activated dendritic cells (CD1+CD80+ rate and MFI, *p* = 0.0173) and activated M2 macrophages (CD163+CD80+ MFI, *p* = 0.0176, and CD163+Cd40+ cell rate, *p* = 0.059) compared to T0 baseline.

#### *Effect of* L. casei *DG® supplementation on the mucosal-adherent microbiota*

**Figure 4.**
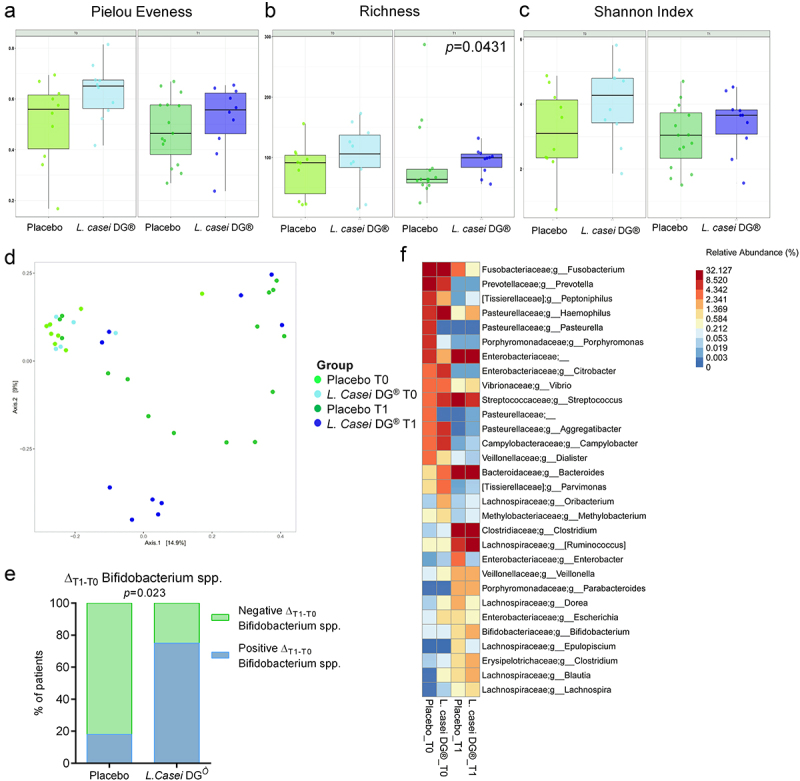
Mucosa-adherent microbiota in the pouch. Box plot of T0 and T1 α-diversity in placebo and *L. casei DG* treated patients at the ASV level according to Pielou Evenness (a), richness (b), and Shannon Index (c). Plot of Bray-Curtis β-diversity Index at T0 and T1 in terms of ASV according to treatment (d). (e) Frequency of patients with positive and negative delta T1-T0 of *Bifidobacterium* spp. In the two arms of the study. (f) Relative abundance of the 30 most abundant genera, presented according to randomization group at T0 and T1.

At T1, the alpha [intra-sample] diversity of samples from the *L. casei DG®*-supplemented group was significantly higher compared to the placebo group as evaluated by using the species Richness index (*p* = 0.043) (Figure 4b). As expected, Bray–Curtis β-diversity showed a significant difference between ileostomy closure (T0) and 8 weeks after ileostomy (T1) mucosal samples, independently from the treatment (Figure 4d). However, no difference was observed in terms of the treatment group, both at T0 and T1 (data not shown). Interestingly, the differential relative abundance analysis identified 65 differentially abundant taxa among treatment groups at T1 (Supplementary data file 1). Notably, *Bifidobacterium* spp. resulted significantly more abundant in the *L. casei DG®*-supplemented group compared to the placebo group, and the *L. casei DG®* supplemented group had a higher frequency of patients with an increase of *Bifidobacterium* spp. in the ileal pouch mucosa in the T1–T0 time frame compared to the placebo group (*p* = 0.023) (Figure 4e). The 30 most abundant genera are shown in Figure 4f, presented according to randomization group at T0 and T1.

#### *Clinical effects of* L. casei *DG® supplementation*

**Figure 5.**
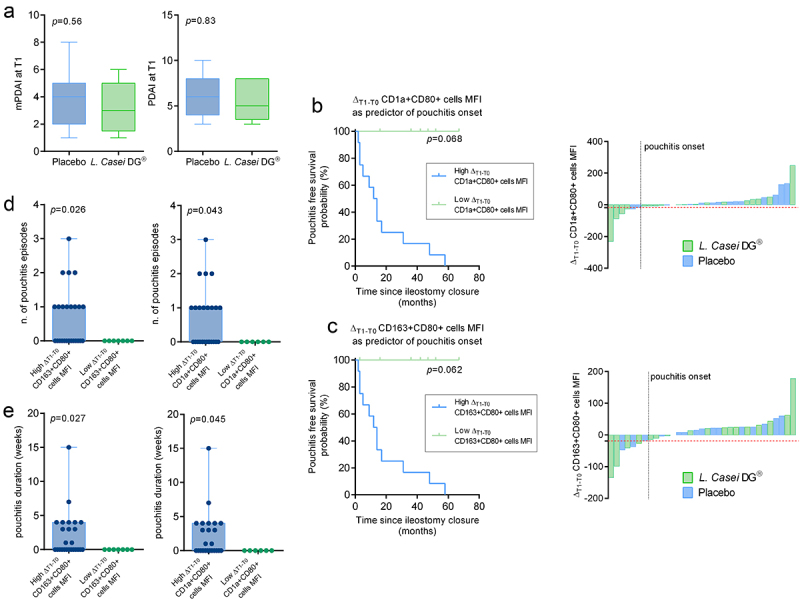
Clinical effect of *L. casei DG* supplementation. (a) PDAI and mPDAI at T1 in the two arms of the study (b) high Δ_T1-T0_ CD1a+Cd80+ and (c) Δ_T1-T0_CD163+Cd80+ cell MFI levels are associated with pouchitis onset. Kaplan-Meier survival curves of high and low Δ_T1-T0_ CD1a+Cd80+ and CD163+Cd80+ cell MFI. (d) Number of pouchitis episodes and of (e) pouchitis duration (weeks) in patients with high and low Δ_T1-T0_ CD1a+Cd80+ and CD163+Cd80+ cell MFI.

No patients showed any sign of clinical pouchitis at ileostomy closure and no difference both in terms of Pouchitis Disease Activity Index (PDAI) and modified PDAI (mPDAI, which omits the histology component) was observed between the two treatment groups at T1 (Figure 5a). Patients were followed up for a median 33.5 (IQR: 13.5–58.5) months. Interestingly, Δ_T1-T0_ CD1a+Cd80+ (dendritic cells) and Δ_T1-T0_ CD163+Cd80+ (M2 macrophages) cells MFI tended to predict pouchitis onset during the follow-up (*p* = 0.068 and *p* = 0.062, respectively) (Figure 5b,c); moreover, patients with high increase of CD1a+Cd80+ and CD163+Cd80+ cell MFI levels in the T0–T1 interval experienced pouchitis more frequently and for longer periods (Figure 5d,e). Notably, as shown in Figure 3c, *L. casei DG®* supplementation was associated with a negative or null variation of activated macrophages and dendritic cells MFI from baseline, while the placebo group showed a significant increase in MFI levels.

### Compliance

At T1 and at T2, at the endoscopy patients were interviewed also about side effect and compliance to the protocol (as measured by the number of days when they effectively took *L. casei DG®* supplementation or the placebo). Probiotics were well tolerated, and the patients reported no mild or serious adverse effects or were recognized by the physicians. Compliance was high (greater than 95%) and no difference in term of compliance was observed between the *L. casei DG®* supplementation or the placebo groups.

## Discussion

In the present work, we carried out a double-blind, placebo-controlled trial of *L. casei DG®* therapy vs. placebo starting at the time of ileostomy closure to evaluate the impact of a change in microbiota on pouch mucosa inflammatory markers and local microenvironment and on consequent pouch outcome. Fifty-two patients were examined to determine the effect of the *L. casei DG*® supplement compared to a placebo. No difference in pouchitis episodes was observed between the two treatment groups while chronic pouchitis onset could not be assessed due to the short-term follow-up. However, the probiotic group showed a significant reduction of TNF-α and IL-6 levels compared to T0 baseline levels in the pouch mucosa, whereas in the placebo group cytokines levels resulted stable. Moreover, the *L. casei* DG®-supplemented group had a significantly higher microbial alpha diversity and probiotic species abundance than the placebo group at T1. Since shifts in the microbiome can influence mucosal immune responses and mucosal pathophysiology,^24^ these findings suggest a reduced risk of developing inflammation. Finally, in the *L. casei* DG®-supplemented group was observed an augmented frequency of patients with reduced variation from baseline of activated dendritic cells in the ileal pouch mucosa compared to the placebo group. Since Landy *et al* had observed that in UC patients with pouchitis dendritic cells were activated,^25^ our finding suggests an anti-inflammatory effect of *L. casei* DG® in the ileal pouch. Our results are in line with recent meta analysis showing that probiotics are effective in preventing pouchitis and that antibiotics are likely effective in treating active pouchitis, suggesting that primarily the modification of ileal pouch microbiota can beneficially affect the fate of the pouch.^11,26^

The study enrollment started in 2016 and lasted until 2022, and was delayed because the study is monocentric and patients were followed up in-house. Moreover, starting from 2020 we encountered several logistic difficulties caused by the COVID pandemic. During the pandemic, we almost stopped operating UC patients if they were not severe. Moreover, even after the first year, in a personnel shortage due to nurse and anesthesiologist displacement in other departments dedicated to COVID patients, we had to give priority to oncological patients and to severe or moderate IBD patients, and thus, ileostomy closure was procrastinated well beyond the usual three months period. These are the reasons for a relatively small trial to last so long.

According to research conducted in a mouse model of intestinal infection, the continuous *L. casei* administration, before and after *Salmonella* challenge, protected the host by modulating the inflammatory response, decreasing TNFα.^27^ Moreover, in a mouse model of small bowel damage induced by fluorouracil, *L. casei* reduced 5-FU-induced inflammation in the colon and small intestine decreasing TNF-α, IL-1β, and IL-6.^28^ These mouse models and our data suggest that there is a direct effect of *L. casei* on inflammation in the J-pouch. Moreover, a Chinese study observed that *L. casei LH23* can significantly ameliorate dextran sulfate sodium-induced mouse colitis *in vivo* by reducing the number of macrophages and their secreted inflammatory cytokines.^29^ *L. casei* is recognized via Toll-like receptor 2 (TLR2) and nucleotide-binding oligomerization domain-containing protein 2 (NOD2) receptors and stimulates bone marrow-derived dendritic cells to produce cytokines in species- and strain-dependent manners.^30^ These findings are consistent with our results and suggest that *L. casei* may effectively mitigate inflammatory infiltration by dendritic cells and macrophages within the ileal mucosa.

It is well established that the microbiota of the pouch changes over time and these modifications can arise as early as two months after surgery and achieve a more stable composition as the years go by.^31^ Indeed, our data confirm a significant difference in β-diversity in mucosa-adherent microbiota between ileostomy closure and 8 weeks after ileostomy, independently from the treatment. Moreover, it has been shown that patients experiencing pouchitis exhibit a lower bacterial diversity.^32,33^ Notably, in our series the *L. casei DG*®-supplemented group had a significantly higher microbial alpha diversity and abundance of species with probiotic properties compared to the placebo group at T1. These results indicate that *L. casei DG®* supplementation profoundly modifies the ileal pouch microbiota inducing protective changes against pouchitis and this is probably the mechanism of its conclaimed anti-inflammatory effect.

No direct clinical effect was observed in *L. casei DG®* supplementation group compared to placebo. This is in line with a previous study showing that short-term probiotics therapy has limited effectiveness and that the average severity of pouchitis and the number of patients with pouchitis significantly decrease after 9 months of probiotics taking.^11^ Indeed, the primary objective of the study was the characterization of the ileal pouch mucosa inflammatory environment after probiotics intake: we chose these parameters to have a more reliable short-term assessment of local inflammation within the pouch mucosa than the mere histological assessment that, in our previous studies, showed a low specificity.^8–10^ Moreover, we could observe that *L. casei DG®* supplementation was associated not only with diminished inflammatory cytokines expression but also with a negative or null difference in activated dendritic cells compared to baseline T0. Thus, the clinical relevance of the study is linked to the implication of its results. We demonstrated that manipulating the ileal pouch microbiota just after the ileostomy closure led to a significant change of the immune microenvironment of the ileal wall. This information may lead to the design of further trials that explore how this manipulation or, more extended ones, may lead to deeper changes that they may finally lead to a significant decrease of pouchitis episodes.

The MEP1 trial is the first randomized controlled trial to have investigated the effect of *L. casei DG®* supplementation on the pouch mucosa microenvironment, but some limitations should be noted. The main limit is the relatively small sample size of the study groups that prevented us to obtain direct clinical indication. The sample size was calculated on bio humoral outcomes and not on clinical ones and to draw direct clinical conclusion probably more studies will be necessary. Moreover, a second limit could be the short supplementation time. Probably a longer supplementation could have enhanced the effects on these patients making possible drawing more meaningful clinical conclusions. Finally, COVID pandemic extremely prolonged the study time because while proctocolectomy for complicated ulcerative colitis continued to be performed almost as usual, ileostomy closure had a very low priority in a restricted setting of reduced resources for surgery.

In conclusion, our study suggests that *L. casei DG®* supplementation just after ileostomy closure, despite the physiologically high bowel movement rate, can effectively impact inflammatory cytokine network and innate immunity effector cell activation within the mucosa of the ileal pouch. Moreover, *L. casei DG®* supplementation profoundly modified the ileal pouch microbiota inducing protective changes against pouchitis and this might be one of the mechanisms of its anti-inflammatory effect. Further investigation is needed to examine whether longer supplementation and larger sample size may show a direct clinical effect of *L. casei DG®* on pouchitis onset.

## Supplementary Material

Supplemental Material

## Data Availability

The data that support the findings of this study are available on reasonable request from the corresponding author. Raw data files for 16S sequencing are available at NCBI Sequence Read Archive (SRA) database under accession number PRJNA1130449.
