# Supplementary material for: Oral administration of Lactobacillus casei DG® after ileostomy closure in restorative proctocolectomy: a randomized placebo-controlled trial (microbiota and immune microenvironment in pouchitis -MEP1)
Source: Gut Microbes. 2024 Nov 1;16(1):2423037. doi: 10.1080/19490976.2024.2423037 (PMC11540070; doi:10.1080/19490976.2024.2423037)
Supplement: Supplemental Material [file KGMI_A_2423037_SM9001.zip › KGMI 2423037/CONSERVE 2020 Checklist MEP1.docx]

CONSERVE Checklists

| **CONSERVE-CONSORT Extension:** | | | | | |
| --- | --- | --- | --- | --- | --- |
| **Item** | **Item Title** | **Description** | | | **Page No.** |
| I. | Extenuating Circumstances | Describe the circumstances and how they constitute extenuating circumstances. | | | Page 13 |
| II. | Important Modifications | a. Describe how the modifications are important modifications. | | | Page 13 |
|  |  | b. Describe the impacts and mitigating strategies, including their rationale and implications for the trial. | | | (see below) |
|  |  | c. Provide a modification timeline. | | | Page 13 |
| III. | Responsible Parties | State who planned, reviewed and approved the modifications. | | | N/A |
| IV. | Interim data | If modifications were informed by trial data, describe how the interim data were used, including whether they were examined by study group, and whether the individuals reviewing the data were blinded to the treatment allocation. | | | N/A |
| **CONSORT Number and Item** | | For each row, if important modifications occurred check “direct impact” and/or “mitigating strategy” and describe the changes in the trial manuscript or supplement. Check “no change” for items that are unaffected in the extenuating circumstance. | | | **Page No.** |
|  |  | **No Change** | **Impact*** | **Mitigating Strategy**** |  |
| 1 | Title and abstract | X |  |  |  |
| 2 | Introduction | X |  |  |  |
| 3 | Methods: Trial Design | X |  |  |  |
| 4 | Methods: Participants | X |  |  |  |
| 5 | Methods: Interventions | X |  |  |  |
| 6 | Methods: Outcomes | X |  |  |  |
| 7 | Methods: Sample Size | x |  |  |  |
| 8-10 | Methods: Randomisation | X |  |  |  |

| 11 | Methods: Blinding | x |  |  |  |
| --- | --- | --- | --- | --- | --- |
| 12 | Methods: Statistical methods | x |  |  |  |
| 13 | Results: Participant flow | X |  |  |  |
| 14 | Results: Recruitment |  | X | X | Page 11 |
| 15 | Results: Baseline data | X |  |  |  |
| 16 | Results: Numbers analysed | X |  |  |  |
| 17 | Results: Outcomes and estimation | X |  |  |  |
| 18 | Results: Ancillary analyses | X |  |  |  |
| 19 | Results: Harms | X |  |  |  |
| 20 | Discussion: Limitations |  | X |  | Page 14 |
| 21 | Discussion: Generalisability | X |  |  |  |
| 22 | Other information: Registration | X |  |  |  |
| 23 | Other information: Protocol | X |  |  |  |
| 24 | Other information: Funding | X |  |  |  |
| *Aspects of the trial that are directly affected or changed by the extenuating circumstance and are not under the control of investigators, sponsor or funder.  **Aspects of the trial that are modified by the study investigators, sponsor or funder to respond to the extenuating circumstance or manage the direct impacts on the trial.  The CONSERVE-CONSORT Checklist is licensed by the CONSERVE Group under the Creative Commons Attribution-NonCommercial-NoDerivs 4.0 International license. | | | | | |
