# Supplementary material for: Oral administration of Lactobacillus casei DG® after ileostomy closure in restorative proctocolectomy: a randomized placebo-controlled trial (microbiota and immune microenvironment in pouchitis -MEP1)
Source: Gut Microbes. 2024 Nov 1;16(1):2423037. doi: 10.1080/19490976.2024.2423037 (PMC11540070; doi:10.1080/19490976.2024.2423037)
Supplement: Supplemental Material [file KGMI_A_2423037_SM9001.zip › KGMI 2423037/Delta2 checklist MEP1.docx]

| Web appendix \| DELTA^2^ recommended reporting items for the sample size calculation of a randomised controlled trial with a superiority question | |
| --- | --- |
| Page and line numbers  Recommended reporting items where item is reported | |
| Core items | |
| (1) Primary outcome (and any other outcome on which the calculation is based) | P7, L129-130 |
| If a primary outcome is not used as the basis for the sample size calculation, state why | N/A |
| (2) Statistical significance level and power | P9, L219 |
| (3) Express the target difference according to outcome type | P9, L219-220 |
| (a) Binary—state the target difference as an absolute or relative effect (or both), along with the intervention and control group proportions. If both an absolute and a relative difference are provided, clarify if either takes primacy in terms of the sample size calculation | N/A |
| (b) Continuous—state the target mean difference on the natural scale, common standard deviation, and standardised effect size (mean difference divided by the standard deviation) | P9, L219-220 |
| (c) Time-to-event—state the target difference as an absolute or relative difference (or both); provide the control group event proportion, planned length of follow-up, intervention and control group survival distributions, and accrual time (if assumptions regarding them are made). If both an absolute and relative difference are provided for a particular time point, clarify if either takes primacy in terms of the sample size calculation | N/A |
| (4) Allocation ratio | P6, L98 |
| If an unequal ratio is used, the reason for this should be stated | N/A |
| (5) Sample size based on the assumptions as per above | P9, L221 |
| (a) Reference the formula/sample size calculation approach, if standard binary, continuous, or survival outcome formulas are not used. For a time- to-event outcome, the number of events required should be stated | N/A |
| (b) If any adjustments (eg, allowance for loss to follow-up, multiple testing) that alter the required sample size are incorporated, they should also be specified, referenced, and justified along with the final sample size | N/A |
| (c) For alternative designs, additional input should be stated and justified. For example, for a cluster randomised controlled trial (or an individually randomised controlled trial with clustering), state the average cluster size and intracluster correlation coefficient(s). Variability in cluster size should be considered and, if necessary, the coefficient of variation should be incorporated into the sample size calculation. Justification for the values chosen should be given | N/A |
| (d) Provide details of any assessment of the sensitivity of the sample size to the inputs used | N/A |
| Additional items for grant application and trial protocol | |
| (6) Underlying basis used for specifying the target difference (an important or realistic difference) | P9, L220 |
| (7) Explain the choice of target difference—specify and reference any formal method used or relevant previous research | P9, L220 |
| Additional item for trial results paper | |
| (8) Reference the trial protocol | P6, L106 |
| This set of reporting items has been developed with the conventional statistical (Neyman-Pearson) approach to a sample size calculation in mind. Some of the reporting items would differ if another approach were to be used. This checklist has been taken from table 1 in *BMJ* 2018;363:k3750, as a standalone document for readers to print out or fill in electronically. | |
