# Supplementary material for: Oral administration of Lactobacillus casei DG® after ileostomy closure in restorative proctocolectomy: a randomized placebo-controlled trial (microbiota and immune microenvironment in pouchitis -MEP1)
Source: Gut Microbes. 2024 Nov 1;16(1):2423037. doi: 10.1080/19490976.2024.2423037 (PMC11540070; doi:10.1080/19490976.2024.2423037)
Supplement: Supplemental Material [file KGMI_A_2423037_SM9001.zip › KGMI 2423037/Supplementary Table 1.docx]

**Supplementary Table 1. Antibodies used in the study**

| **Target** | **Conjugate** | **Clone** | **Company** |
| --- | --- | --- | --- |
| CD1a | PE | HI149 | ImmunoTools GmbH |
| CD4 | PE-Cy7 | RPA-T4 | eBioscience |
| CD8 | FITC | RPA-T8 | eBioscience |
| CD40 | FITC | 5C3 | eBioscience |
| CD69 | PE | FN50 | eBioscience |
| CD80 | PE-Cy7 | 2D10.4 | eBioscience |
| CD163 | PE | GHI/61 | eBioscience |
